# Implementation of a stepped wedge cluster randomized trial to evaluate a hospital mobility program

**DOI:** 10.1186/s13063-020-04764-7

**Published:** 2020-10-16

**Authors:** Susan N. Hastings, Karen M. Stechuchak, Ashley Choate, Elizabeth P. Mahanna, Courtney Van Houtven, Kelli D. Allen, Virginia Wang, Nina Sperber, Leah Zullig, Hayden B. Bosworth, Cynthia J. Coffman

**Affiliations:** 1 Center of Innovation to Accelerate Discovery and Practice Transformation, Durham VA Health Care System, Durham, NC USA; 2 Geriatrics Research, Education and Clinical Center, Durham VA Health Care System, Durham, NC USA; 3 Department of Medicine, Duke University School of Medicine, Durham, NC USA; 4 Department of Population Health Sciences, Duke University School of Medicine, Durham, NC USA; 5 Center for Aging, Duke University School of Medicine, Durham, NC USA; 6 Department of Medicine and Thurston Arthritis Research Center, University of North Carolina, Chapel Hill, NC USA; 7 Department of Biostatistics and Bioinformatics, Duke University School of Medicine, Durham, NC USA

**Keywords:** Stepped wedge, Mobility, Pragmatic trials

## Abstract

**Background:**

Stepped wedge cluster randomized trials (SW-CRT) are increasingly used to evaluate new clinical programs, yet there is limited guidance on practical aspects of applying this design. We report our early experiences conducting a SW-CRT to examine an inpatient mobility program (STRIDE) in the Veterans Health Administration (VHA). We provide recommendations for future research using this design to evaluate clinical programs.

**Methods:**

Based on data from study records and reflections from the investigator team, we describe and assess the design and initial stages of a SW-CRT, from site recruitment to program launch in 8 VHA hospitals.

**Results:**

Site recruitment consisted of thirty 1-h conference calls with representatives from 22 individual VAs who expressed interest in implementing STRIDE. Of these, 8 hospitals were enrolled and randomly assigned in two stratified blocks (4 hospitals per block) to a STRIDE launch date. Block 1 randomization occurred in July 2017 with first STRIDE launch in December 2017; block 2 randomization occurred in April 2018 with first STRIDE launch in January 2019. The primary study outcome of discharge destination will be assessed using routinely collected data in the electronic health record (EHR). Within randomized blocks, two hospitals per sequence launched STRIDE approximately every 3 months with primary outcome assessment paused during the 3-month time period of program launch. All sites received 6–8 implementation support calls, according to a pre-specified schedule, from the time of recruitment to program launch, and all 8 sites successfully launched within their assigned 3-month window. Seven of the eight sites initially started with a limited roll out (for example on one ward) or modified version of STRIDE (for example, using existing staff to conduct walks until new positions were filled).

**Conclusions:**

Future studies should incorporate sufficient time for site recruitment and carefully consider the following to inform design of SW-CRTs to evaluate rollout of a new clinical program: (1) whether a blocked randomization fits study needs, (2) the amount of time and implementation support sites will need to start their programs, and (3) whether clinical programs are likely to include a “ramp-up” period. Successful execution of SW-CRT designs requires both adherence to rigorous design principles and also careful consideration of logistical requirements for timing of program roll out.

**Trial registration:**

ClinicalsTrials.gov NCT03300336. Prospectively registered on 3 October 2017.

## Background

The stepped wedge cluster randomized controlled trial (SW-CRT) design has been recommended as a useful tool for evaluating innovations in health care delivery [1]. In SW-CRTs, clusters are randomized to the order in which they become exposed to an experimental condition. As such, timing is a critical element of both design and successful execution of these types of trials. This design increasingly appeals to learning health systems that desire to conduct pragmatic evaluations of intervention effects under the usual conditions in which the intervention will be applied. The Veterans Health Administration (VHA), as a large integrated health care system with a fully implemented electronic health record and mature embedded research infrastructure, is well-suited for conducting pragmatic evaluations [2] using SW-CRTs.

SW-CRT designs have been mainly applied to evaluate interventions that have been shown to be effective in previous research in other settings and/or where there is a strong belief that dissemination of the intervention will do more good than harm [3]. One example of an area with this type of “implementation momentum” [4] is hospital mobility. Hospital mobility interventions have demonstrated the potential to prevent functional decline and to reduce hospital lengths of stay, leading to growing interest from health systems [5–9], including VHA. The SW-CRT could provide a useful framework for evaluating the rollout of hospital-based programs, although it is acknowledged to be a complex trial design. Multiple reviews have been published that focus on statistical methodology using SW-CRTs; however, the literature is sparse on how to deal with the complexities around timing for rolling out the intervention under evaluation [10]. There is a particular need for practical guidance on how to apply this design in evaluating dynamic clinical programs that may require new staffing models or changes in workflow for widespread implementation.

We share our experiences conducting the initial stages of a SW-CRT to evaluate a hospital mobility program (STRIDE) in VHA. A SW-CRT design with all sites receiving the intervention was selected as the most efficient approach to achieve our goals of a mixed method evaluation of both implementation and effectiveness. In this paper, we describe the components of the SW-CRT for STRIDE, focusing on decisions related to trial design and their impact on the study to date. We present lessons learned from STRIDE recruitment through the time of program launch and provide recommendations for design considerations for future studies utilizing SW-CRTs to evaluate clinical programs in hospitals. These considerations represent some key points that may be overlooked and, if not considered carefully, may jeopardize successful program implementation and study completion.

## Methods

### Study design overview

We describe a SW-CRT that is ongoing in 8 VHA hospitals as part of the Optimizing Function and Independence Quality Enhancement Research Initiative program (Function QUERI) [11]. STRIDE is an evidence-based supervised walking program for hospitalized older adults designed to reduce the negative consequences of immobility [12, 13]. The program consists of a one-time gait and balance assessment conducted by a physical therapist, followed by daily supervised walks led by a therapy or nursing assistant for the duration of the hospital stay. STRIDE was developed and tested at the Durham VA Health Care System (VA HCS) as a clinical demonstration program in 2012. It was designed for patients 60 years old and above, the demographic group most susceptible to functional decline and other negative consequences of immobility in the hospital [14]. In the initial evaluation, patients receiving STRIDE were more likely to be discharged to home than skilled nursing or rehabilitation facilities compared to clinically similar patients receiving usual care (92% vs. 74%, *p* = 0.007) and had a reduced length of stay by 1 day, consistent with findings from other similar hospital mobility programs [12, 15].

**Fig. 1:**
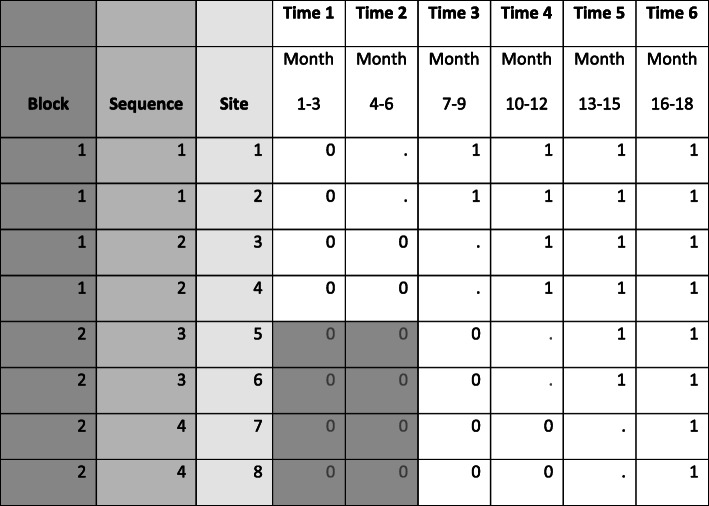
Originally proposed blocked SW-CRT design for Function QUERI STRIDE. “0” indicates pre-implementation period, “.” indicates implementation period (STRIDE launch), “1” indicates post-implementation period, and “0” in shaded gray indicates prior to VA hospital recruitment for block 2 where primary outcome data for control time periods can still be collected via EHR (but not patient-reported data in these blocks because the sites have not enrolled in the study during this time period)

An overview of our original study design is presented in Fig. 1. In a SW-CRT, clusters (in this case hospitals) are randomized to one of several different implementation schedules, referred to as sequences, which indicate the time at which the cluster will switch (cross over) from the control condition to the intervention condition [1]. The SW-CRT design results in a staggered start which can help to address a common challenge of having insufficient resources to roll out the intervention simultaneously to all clusters [16].

Three important early and related design decisions regarding the timing of our study were (1) whether to conduct a single randomization or randomize in blocks, (2) how many clusters would be randomized within each sequence, and (3) how long each time period would last.

In the Function QUERI STRIDE SW-CRT, we enrolled and randomized hospitals to their sequence in 2 distinct blocks (as shown in Fig. 1). We did not assign sites to one block or the other. Block 2 sites were recruited separately after block 1 sites had been randomized and assigned to their sequence. The primary motivation for this decision was to reduce the time sites were asked to wait to begin their programs and thereby enhance site engagement and retention. If we had recruited, enrolled, and randomized all 8 sites at once, the 2 hospitals assigned to the 4th and final sequence would have been asked to wait a full year before starting their programs. We were concerned that due to inactivity with implementing the program for this length of time along with the changing dynamics in health care systems (e.g., change in management, strategic goals) that these hospitals might lose interest in the STRIDE program and would drop out or initiate the program on their own, prior to their assigned launch window.

We randomized 2 hospitals (clusters) to each sequence, meaning 2 hospitals were assigned the same start date and overall study schedule. A main consideration in this decision was estimating how many hospitals we could support in both implementation and data collection activities at the same time based on available study staff. We selected 3 months as the duration of each time period because we estimated this would give hospitals a reasonable window of time in which to launch their programs and also be long enough to assess the impact of intervention on our primary outcome (discharge destination). The duration of the time period along with the number of clusters per randomization sequence determines how many time periods are needed to complete the SW design to conduct the trial and this can have implications for other design decisions (e.g., number of subjects, overall length of study).

In Function QUERI STRIDE, hospitals were eligible to participate if they had a minimum average daily census of 20 general medicine patients per day (approximately 250 in a 3-month window) and agreed to start a STRIDE program within their randomly assigned 3-month window for program launch. The primary outcome, discharge destination (home vs. other), will be assessed at the patient-hospitalization level using routinely collected data in the electronic health record (EHR) and health care claims data. Other clinical outcomes include length of stay and patient-reported measures such as self-rated physical and psychosocial function, collected via telephone survey in a subset of participants. A description of the mixed methods evaluation plan has been previously published [11]. STRIDE was implemented at participating sites as a clinical program; evaluation was approved as human subject research by the Durham VA Institutional Review Board (protocol #02040).

In the following sections we describe our experiences with implementing the initial stages of the trial. Based on data from study records and reflections from the investigator team, we present our experiences from site recruitment to program launch and implications for design decisions in future SW-CRTs.

## Results

### Experiences with site recruitment and enrollment

Table 1 summarizes some key Function QUERI STRIDE SW-CRT design decisions and their impact on the study.

**Table 1:** Function QUERI STRIDE SW-CRT design decisions and their impact on the study

Design decision	Function QUERI SW-CRT	Advantages	Disadvantages
Time period length	3 months	Shorter length and larger number of time periods increased power	Longer time period length would have given sites longer window to launch program
Number of sites per sequence	2	Allowed study team to provide significant implementation support to each site; study conducted faster than if only 1 site/time period	Power implications; maximum power with one cluster per sequence
Recruitment and randomization strategy	Recruited and randomized clusters to sequences in 2 separate blocks	Reduced maximum possible time from enrollment to program launch time; ability to start study before all sites recruited; potentially fewer dropouts; ability for earlier sites to serve as mentors for later sites	Not able to collect patient-reported data from block 2 sites in time periods before enrollment Potential confounding of treatment with block Full statistical implications of blocked randomization for SW-CRT have not been studied. Calendar time versus time since randomization may be an issue for blocks
Complete vs. incomplete design and type	Incomplete design—outcome data during implementation interval not used for evaluation	Avoided including data when program is partially implemented	Power implications; loss of data from time periods not included in evaluation

#### Recruitment

**Fig. 2:**
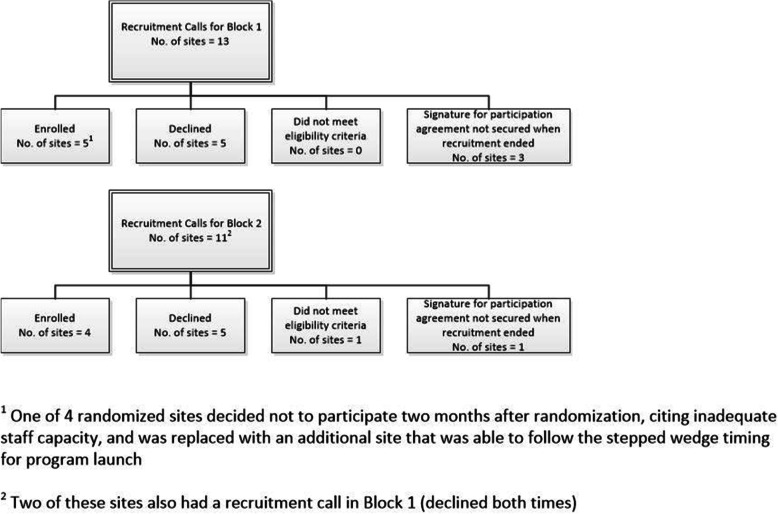
Function QUERI STRIDE SW-CRT site recruitment and enrollment

Figure 2 depicts the flow of site recruitment and enrollment. We advertised the study through multiple means including calls with national VA leaders and staff, presentations at clinical conferences and other meetings, and newsletters. We had thirty 1-h conference calls with representative(s) from 22 VAMCs who expressed interest in implementing STRIDE; 2 of these VAMCs had conference calls for both recruitment blocks and were considered as separate sites in reporting recruitment numbers. Of these, 10 declined to participate and 1 did not meet inpatient census eligibility requirements. The most common reasons for refusal were inadequate staff capacity, unable to obtain key leadership support, or competing priorities with other programs and initiatives. For block 1, of the 13 sites interested, we enrolled and randomized the first 4 to return completed participation agreements by July 2017. In block 1, one site decided not to participate 2 months after randomization, citing inadequate staff capacity, and was replaced with an additional site that was able to follow the SW timing for program launch (no data are presented for the site that withdrew in the early phase of study start-up). Delayed entry of the replacement site into the SW design shortened the pre-exposure period by approximately 3 months (1 time period) for data collected via telephone survey, but did not impact EHR primary outcome data collection. For block 2, of the 11 sites interested, we enrolled and randomized the first 4 to return completed participation agreements by April 2018.

Site characteristics for the 8 participating sites are presented in Table 2. In block 2, half of participating hospitals had the highest complexity level compared to 3 out of 4 in block 2. Block 1 sites had a higher turnover rate among registered nurses (mean 5.1 compared to 2.8). Other performance and quality metrics that we examined were similar.

**Table 2:** Characteristics of enrolled hospitals

	Total (***N*** = 8)	Block 1 sites (***n*** = 4)	Block 2 sites (***n*** = 4)
Hospital 5 star performance rating^a,b,d^, mean (SD)	3.5 (0.8)	3.5 (1.0)	3.5 (0.6)
Facility complexity level^c,d^, *n* (%)			
1a	5 (62.5)	2 (50)	3 (75)
1b	2 (25)	1 (25)	1 (25)
1c	1 (12.5)	1 (25)	0 (0)
Registered nurse turnover rate %^a,e^, mean (SD)	4.0 (1.8)	5.1 (2.1)	2.8 (0.4)
Adjusted length of stay^a,e^, mean (SD)	4.4 (0.3)	4.2 (0.3)	4.5 (0.3)
Hospital-wide 30-day readmission rate^a,e^, mean (SD)	12.3 (0.8)	12.6 (0.7)	12.0 (0.9)
Overall rating of hospital (inpatient) ^a,e^, mean (SD)	64.2 (5.5)	66.6 (7.2)	61.8 (1.6)
Employee satisfaction with organization^a,e^, mean (SD)	3.6 (0.1)	3.6 (0.1)	3.5 (0.2)
Patient safety indicator^a,e^ mean (SD)	0.1 (0.4)	0.1 (0.5)	0.1 (0.2)
In-hospital complications^a,e^, mean (SD)	1.0 (0.2)	1.0 (0.2)	1.0 (0.2)

*SD* standard deviation

^a^Strategic Analytics for Improvement and Learning (SAIL) measure. SAIL is a system for summarizing hospital system performance within VHA

^b^Hospital 5-star rating (1–5) indicates a VA hospital’s quality of care relative to other VA hospitals and is based on data such as death rates, nursing turnover, patient satisfaction, and efficiency [17]

^c^Facility complexity level classifies VHA facilities at levels 1a, 1b, 1c, 2, or 3 with level 1a being the most complex and level 3 being the least complex. The model is reviewed and updated with current data every 3 years. The peer grouping system is based on seven variables relating to patient population, clinical services complexity, and education and research [18]

^d^Rating from fiscal year 2017

^e^Rating from quarter 3 in fiscal year 2017

#### Randomization strategy

**Fig. 3:**
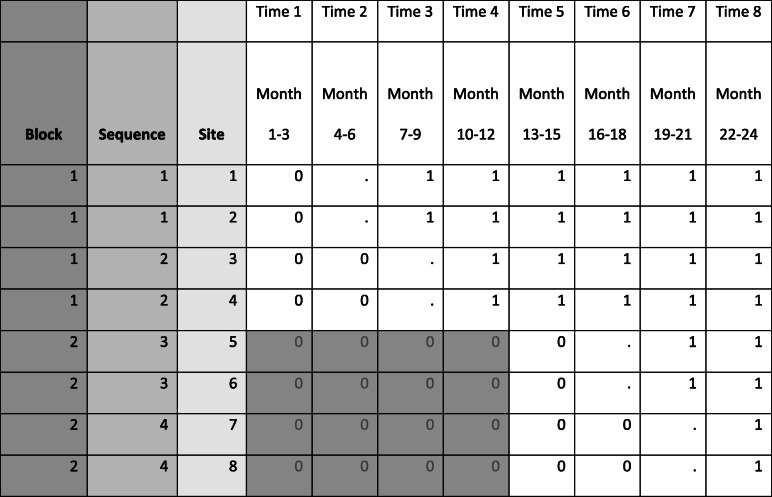
Implemented blocked SW-CRT design for Function QUERI STRIDE with 6-month delay in recruitment of block 2 sites. “0” indicates pre-implementation period, “.” indicates implementation period (STRIDE launch), “1” indicates post-implementation period, and “0” in shaded gray indicates prior to VA hospital recruitment for block 2 where primary outcome data for control time periods can still be collected via EHR (but not patient-reported data in these blocks because the sites have not enrolled in the study during this time period)

Due to the small number of clusters in our trial, a potential benefit of randomization may be lost as the balance of known and unknown confounders may not be possible [19]. Therefore, as we weighed the risk of a blocked randomization with a potential loss of a cluster or clusters, we felt that moving forward with the blocked randomization was a smaller risk. Our initial intention with the 2-block design was to adhere to the same schedule as if all 8 hospitals had been recruited and randomized at the same time as shown in Fig. 1. However, as shown in Fig. 3, the 2nd phase of recruitment was delayed by 6–7 months. Therefore, instead of the first sequence in block 2 launching the program during time period 4 (as indicated in Fig. 1), the launch occurred in time period 6 (2 time periods later than intended). We were able to address this delay by extending the study from 18 to 24-months, adding 2 time periods to each block for data collection to complete the SW design (Fig. 3).

### Experiences with program launch

#### Implementation support and timing of program launch

The eight hospitals implemented their STRIDE programs following the blocked randomized stepped wedge design according to 4 sequences over a total of 24 months. As noted earlier, an important consideration in determining the number of hospitals assigned to each sequence was the amount of study resources needed to support sites in achieving program launch within their assigned window. The Function QUERI team used the Replicating Effective Programs (REP) framework to support each site team in developing and launching their STRIDE program [11]. REP consists of a series of activities to support implementation of the core elements of a program that are essential for fidelity, while allowing space for stakeholder input and flexibility to tailor non-core aspects of the program to site-specific resources [4, 20–22]. All sites received 6–8 implementation support calls, according to a pre-specified schedule, from the time of recruitment to program launch [13]. All sites identified an internal primary point of contact (POC) to work with the Function QUERI research team and champion the implementation of STRIDE in their facility. The POC convened an interdisciplinary team to participate in REP activities, including a series of scheduled calls and one in-person site visit by the Function QUERI team over the course of 3 months prior to program launch.

With each subsequent sequence, program launches went more smoothly as the implementation team honed the content of support calls and became more adept at assisting hospital teams with problem-solving. Study implementation specialists reported that when a site assigned to a later program launch date reported a challenge, they were able to share lessons learned from prior site’s implementation or facilitate communication between participating sites for counsel. These learnings had to do with tips on how to get the program off the ground, for example, programming progress notes for the electronic health record. The core elements of the clinical program were held constant across sequences, consistent with the REP framework.

#### Program ramp-up

The Function QUERI STRIDE SW-CRT has an incomplete design, meaning that for each sequence, the evaluation does not include primary outcome data during the 3-month time period devoted to starting the program (indicated as the “implementation period” in Figs. 1 and 3). These outcome data are not used in the evaluation to account for the fact that sites are not likely to have their programs operating at full capacity on the first day of launch and to give some flexibility to sites to set the timing of the launch within the 3-month window. All 8 sites successfully launched within their assigned 3-month implementation window, with variation across sites: three sites launched in the first month, 2 in the second month, and 3 in the last month of their implementation window. Seven of the eight sites started with a limited or modified version of their program during the implementation time period, 6 sites limited the number of medicine wards that offered the program with plans to expand to the remaining medicine wards after they presented initial results to leadership, and one site initially limited the program to include only patients who also received a certain type of consult. Three sites implemented the program with existing staff before hiring a dedicated walk assistant.

## Discussion

Pragmatic trials that examine the rollout of new programs using existing staff and resources are needed to help accelerate the adoption of evidence-based interventions [23]. SW-CRTs can be valuable tools for evaluating innovations in learning health care systems, yet there are many challenges in executing these types of studies. Based on our early experiences utilizing a SW-CRT to evaluate implementation of a new clinical program in VHA hospitals, we highlight several key design decisions that researchers need to consider for future studies. We also report unexpected advantages of this design that may be harnessed for future implementation research. These recommendations should be considered in conjunction with other resources that offer guidance on whether a SW-CRT design is appropriate [24] and how to address analytical challenges [25] and ensure comprehensive reporting of completed SW-CRT trials [1].

First, does a blocked randomization meet study needs? In our study, we believe the blocked randomization enhanced site recruitment and retention and allowed for more efficient allocation of study staff time. If we recruited and randomized all eight hospitals at once, program rollout at hospitals in the last sequence would not have started until a full year after randomization. Given that interested sites were motivated to improve hospital mobility, we were concerned that this long of a wait would deter participation. Therefore, we decided to stagger the recruitment and randomization into 2 blocks with 2 sequences each. The potential benefits of a non-standard randomization must be counterbalanced by potential confounding of blocks. In our study design, blocked randomization had no impact on power because our primary outcome is drawn from routinely collected data in the EHR. We are able to collect outcome data from hospitals in block 2 for the time periods before they were actively enrolled. A blocked randomization may not be feasible for studies that rely on purposively collected data for their evaluation, rather than retrospective data available within the EHR. Highlighting this point, in our study blocked randomization did come at a cost of some data that were collected by telephone for assessing secondary outcomes in block 2 sites. Site recruitment was time-intensive—another advantage of the blocked randomization was the ability to start the study without having to wait for all sites were enrolled, which was more efficient for optimal use of study staff resources. Overall, we found definite logistical advantages to a blocked randomization in this SW-CRT; however, due to the confounding of block with treatment, analytical models will need to include an adjustment for block. It also may be the case that calendar time compared to time since randomization maybe an issue with the block design as all sites are not randomized at the same time. The full implications of a blocked randomization from a statistical perspective require further study.

Second, how much time and implementation support will sites need to launch their programs? In our study, the primary outcome (discharge destination) is assessed very soon after the intervention; therefore, the primary driver of time period length was estimating the window hospitals would need to start their programs. Determining time period length is a critically important decision in a SW-CRT [26], because time period duration along with the number of clusters per randomization sequence determines how many time periods are needed to conduct the trial, which in turn influences power as well as the overall time it takes to conduct the study [26]. Time is also important for providing implementation support. In this study, we utilized the SW-CRT to evaluate both effectiveness and implementation aims. Thus, we had an a priori focus on delivering implementation support to each site to promote program adoption. It is increasingly recognized that the use of evidence-based practices rarely occurs automatically and effective strategies to implement interventions into clinical practice are necessary to ensure that patients receive the benefits [27]. Therefore, it is critical to estimate the time and staffing resources needed to support implementation of new programs and factor this into determining number of sites per sequence, i.e., assigned to launch on the same schedule. In the current study, all sites were able to adhere to their assigned time period for clinical program launch according to the randomization schedule, but this required significant support from the study implementation specialists.

Third, will sites begin with a modified version of the program? Some types of interventions can begin on a specific date that is under the control of the study team, for example, activation of a new alert or clinical dashboard within an electronic health record [28]. In our study, participating sites were responsible for providing existing clinical staff or obtaining funding for new staff to implement the program. Implementing new programs such as STRIDE that require coordination between multiple professions (e.g., nurses, physicians, therapists) in the inpatient setting is challenging. Different professions may perceive their roles and work goals related to mobility differently and interprofessional coordination can vary according to hospital structure, priorities, and resources [29]. In this context, it was not realistic to expect hospitals to be ready to start their programs operating at full capacity on a single date assigned at randomization. Therefore, we used an incomplete design during which there was an a priori plan not to include primary outcome data during the implementation time period. This design allowed sites to start their programs with a ramp-up period, during which they were able to test and tailor the program to suit their needs. Others have advocated for suspended assessment during the implementation period to ensure that the evaluation reflects the effects of a full-strength intervention [30].

Finally, we highlight some positive aspects of SW-CRTs specifically for implementation studies. From a pragmatic perspective, in a SW design, aspects of the implementation can be modified or improved as the study team learns from past sites and new sites are activated. As noted earlier, our initial plan was that the STRIDE rollout using the 2-block design would have occurred with the same timing as if all 8 hospitals had been recruited and randomized simultaneously. However, there was a delay in the start of recruitment for block 2. An unexpected advantage of the delay was that it allowed the implementation team time to process their experience with supporting the first 4 sites and improve procedures for supporting the final 4 sites. An important aspect of our mixed method evaluation plan is to explore implementation experience and outcomes over time so that we can further understand this aspect of our study design. Importantly, we did not observe any changes in the intervention itself over time, consistent with our use of the REP implementation framework which is explicitly designed to promote fidelity to core elements of evidence-based programs. Another unanticipated, but welcome occurrence, was the sequential rollout design provided an opportunity for sites randomized to earlier implementation of STRIDE to serve as mentors to later sites. Building on this experience, we have since developed diffusion networks to support both initial implementation and sustainment of STRIDE at hospitals across VHA. The goals are to capture and share local knowledge and create a collaborative environment for peer-to-peer sharing of experiences and best practices to support implementation via regular teleconferences.

## Conclusions

Early experiences executing a SW-CRT pragmatic trial in VHA suggest several key design considerations to evaluate rollout of a new clinical program. Future studies should incorporate sufficient time for site recruitment and carefully consider the following to inform design of SW-CRTs to evaluate roll-out of a new clinical program: (1) whether a blocked randomization fits study needs, (2) the amount of time and implementation support sites will need to start their programs, and (3) whether clinical programs are likely to include a “ramp-up” period. Site enrollment in blocks may help with engagement by reducing the amount of time sites are asked to wait to launch their programs; however, further study is needed to understand all the statistical implications of the blocked randomization. An incomplete SW-CRT design that incorporates pause periods for data collection during implementation is recommended when clinical programs are likely to include a ramp-up period. Implementation studies should consider whether incorporating peer mentoring strategies would be an effective means to leverage the sequential design. In sum, successful execution of SW-CRT designs to evaluate implementation of clinical programs requires both adherence to rigorous design principles and also careful consideration of logistical requirements for timing of program roll out.

## Data Availability

All data generated or analyzed during this study are included in this published article and its supplementary information files.

## Abbreviations

CRT: Cluster randomized trial
EHR: Electronic health record
REP: Replicating Effective Programs
SW-CRT: Stepped wedge cluster randomized trial
VA: Veterans Affairs
VA HCS: Veterans Affairs Health Care System
VAMC: Veterans Affairs Medical Center
VHA: Veterans Health Administration
POC: Point of contact
